# Caregivers in China: Knowledge of Mild Cognitive Impairment

**DOI:** 10.1371/journal.pone.0053928

**Published:** 2013-01-11

**Authors:** Baozhen Dai, Zongfu Mao, John Mei, Sue Levkoff, Huali Wang, Misty Pacheco, Bei Wu

**Affiliations:** 1 School of Management, Jiangsu University, Zhenjiang, People’s Republic of China; 2 Research Institute for New Rural Development of Jiangsu University, Zhenjiang, People’s Republic of China; 3 School of Public Health, Wuhan University, Wuhan, People’s Republic of China; 4 Department of Anthropology, William James Hall, Harvard University, Cambridge, Massachusetts, United States of America; 5 College of Social Work, University of South Carolina, DeSaussure College, Columbia, South Carolina, United States of America; 6 Dementia Care & Research Center, Peking University Institute of Mental Health, Beijing, People’s Republic of China; 7 University of Hawaii at Manoa, Honolulu, Hawai‘i, United States of America; 8 School of Nursing and Global Health Institute, Duke University, Durham, North Carolina, United States of America; Federal University of Rio de Janeiro, Brazil

## Abstract

This study aimed to examine the experience and knowledge of mild cognitive impairment (MCI) among Chinese family caregivers of individuals with MCI. The sample was recruited from memory clinics in Zhongnan Hospital in Wuhan, China. In-depth semi-structured interviews were used. Thirteen family members of individuals diagnosed with MCI participated in the study. Data analysis revealed three themes: 1) initial recognition of cognitive decline; 2) experience of the diagnosis of MCI; 3) perception of cognitive decline as a normal part of aging. While family members recognized the serious consequences of memory loss (e.g. getting lost), they would typically not take their family members to see a doctor until something specific triggered their access to the medical care system. The Chinese traditional perception of dementia as part of normal aging may serve to lessen the stigma of individuals with MCI, while the term “*laonian chidai”* which literally translates to “stupid, demented elderly” may exacerbate the stigma associated with individuals with MCI. It is suggested that family members’ worries may be relieved by improving their access to accurate knowledge of the disease, community-based and institutional care services, and culturally appropriately words are needed for MCI.

## Introduction

The rapid growth of the elderly population in China has caused an increase in the number of individuals with dementia [1]. It is estimated that approximately 5 million people in China suffer from dementia, of which Alzheimer’s disease (AD) is the most common type. There is currently no treatment available to cure AD [2], [3]. Individuals with mild cognitive impairment (MCI), “a syndrome defined as cognitive decline greater than expected for an individual’s age and education level but does not interfere notably with activities of daily life,” are considered at risk for developing dementia, with a reported annual conversion rate of 14.1% among Chinese elderly aged 55 years and above, compared to a conversion rate of 1.6% among similarly aged cognitively normal Chinese older adults [4], [5]. Current estimates of the prevalence of MCI among elders aged 55 years or over in China range from 2.4% to 14.7% [6], [7].

Although MCI can present with a variety of symptoms, it is termed “amnestic MCI (aMCI)” when memory loss is the predominant symptom. Evidence showed that individuals with aMCI would develop to AD at a rate of approximately 10% to 15% per year [8]. Those who have impairments in domains other than memory are classified as non-amnestic MCI, and they are believed to be more likely to convert to other dementias [9].

The large majority of people with dementia (96%) are cared for at home by family members in China [10]. Family members of individuals with MCI are major potential informal caregivers of AD if individuals with MCI develop into AD. While not all individuals with aMCI actually progress to AD, it is not yet known which factors predict whether MCI will worsen over time [2], [3]. Early detection and intervention may hold promise in determining the cause of the syndrome and delaying the onset of AD. It has been suggested that appropriate and effective ways to help family members of individuals with MCI is essential to address the public health impact of AD [11]–[13]. A better understanding of family members’ knowledge of MCI will contribute to developing appropriate and effective interventions and services to address this public health issue. Although there is an extensive literature on family caregivers' knowledge about AD [14]–[17], empirical research on the knowledge of MCI among family caregivers of individuals with MCI are limited, and little empirical information is available to assist individuals with MCI and their family members [13], [18]–[20]. To our knowledge, there is no empirical study conducted on the knowledge of MCI among Chinese family caregivers.

This study is part of a project focusing on Chinese family members of individuals with cognitive impairment with the purpose of identifying information for developing a culturally appropriate follow-up intervention for family members of elderly individuals with cognitive impairment in China. In this paper, we focus on the experiences and knowledge of MCI among family members of individuals with MCI. We believe that such information is necessary to assist policy makers, researchers, and health care providers in creating supportive interventions for family caregivers of individuals with MCI.

## Methods

We relied on in-depth semi-structured interviews with surveys of family members of individuals with MCI. Grounded theory was used to reveal common themes derived from the data itself [21], [22]. This method is useful to describe the similarities among persons with specific experiences. In-depth qualitative interviews were conducted with 13 family members of individual with MCI.

### Sampling

We recruited participants from an epidemiological study on dementia titled “Cognitive Impairment Survey in Chinese Hospitals” which lasted from October to December, 2009, among 42 major hospitals in China, including Zhongnan Hospital, Wuhan. The physicians in the memory clinics at Zhongnan Hospital at Wuhan University in Wuhan, China, referred the individuals with MCI and their family members to the study team immediately after these individuals were being diagnosed. The diagnosis of MCI was adopted from Petersen et al. (1999): 1) memory complaint, preferably corroborated by an informant; 2) objective memory impairment for age; 3) preservation of general cognitive and functional abilities; 4) not demented [23]. The objective memory impairment was measured by Mini-Mental State Examination. Petersen et al (1999) focused on MCI as a prodromal condition for AD and it emphasized memory impairment [23]. Therefore, individuals with MCI referred to the study team were amnestic MCI (aMCI).

Inclusion criteria for family members were: 1) 18 years or above; 2) willing and able to describe the MCI diagnosis experience; and 3) primary family caregiver now or most likely to become the primary family caregiver of individuals with MCI in the near future. One caregiver per family was recruited in the study.

The protocol of the study was approved by the Institutional Review Board (IRB) at Wuhan University Health Sciences Center and verbal informed consent was obtained prior to each interview. Appointments for the face-to-face, in-depth interviews were made by telephone in a week after the individuals with MCI and their family members were referred to the study team. The interviews were conducted according to the interviewee's preference, mainly within two weeks, at home.

Each interview was recorded with a digital voice recorder after the interviewee agreed. All consent procedures above were approved by the IRB. Eighteen patients were referred to the study team, of whom 5 completed the survey questionnaire without in-depth interview, including 2 who terminated the interview prior to completion, 1 who lived alone and had no family caregiver, and 2 who lived with their paralytic family members and had to take care of them. Thirteen participants satisfied the research criteria and completed the in-depth, face-to-face interviews between October and December, 2009.

### Data Collection

A survey questionnaire was completed to collect the demographic data before the interview began. The in-depth interviews lasted 60–180 minutes. The research questions were shaped by the explanatory model [24], [25]. Each interview began with a broad data-generating question: “Please tell me about your experiences with the family member before and since he/she was diagnosed with mild cognitive impairment.” After the participants described their story, additional questions were asked to further understand their concerns and coping strategies related to the MCI diagnosis. Example probes were: “Please tell me about your concerns regarding the diagnosis of MCI.” “What are the causes of memory loss in your opinion?” etc.

Efforts were made to ensure the validity and credibility of the results. The interviewers were given systematic training about how to conduct interviews to ensure a consistent approach in the interview process. Each interview was conducted by two trained researchers who understood the local dialect with one responsible for taking the notes and one responsible for the interviewing. The questions strictly followed the topic guidelines. Supervision of the interview was done by the one who was responsible for taking the notes by checking the topical outline throughout the interview process to ensure that interview was semi-structured and the interviewer followed the topical outline with an adequate coverage of topics. Prior to the interviews, study investigators had conducted a pilot study obtaining process data from family caregivers to ensure the suitability of the open-ended questions.

### Data Analysis

Each interview was recorded with a digital voice recorder. Data were analyzed by a research team. Two research members independently transcribed the recorded tapes into Mandarin Chinese and compared results. For accuracy, the transcriptions were compared again with the recorded tapes by two research team members who understood the local dialect. Another two research members independently translated the transcription into English and compared results with each other. Discrepancies in the translation were resolved by seeking input from senior bilingual researchers and conducting back translation of the transcripts from English to Chinese.

Three coders who had been trained for grounded theory and opening coding independently coded the translations for themes, sub-themes, and categories. Open coding was conducted by three coders with line-by-line coding [22]. The codes were checked by the corresponding author to ensure the validity of the interview coding. Discrepancies in coding were resolved by discussion among the coders. After the consensus of the codes was obtained, the codes were put into categories, from which the themes and sub-themes were extracted. Themes were consolidated to figure out “core variables” present in the majority (i.e., more than 60%) of interviews until saturation was attained and no new theme emerged. Only after the co-authors obtained consensus of the thematic results was the codebook created. Descriptive analysis was conducted, using SPSS 15.0 [26].

## Results

### Participant Characteristics

Thirteen family members of different individuals with MCI participated in the study. The majority of caregivers were female (54%), married (92%), retired (85%), and graduates of high school or higher levels of education (92%), with a mean age of 68.5 (±12.3) years (range from 41 to 85) (Table 1). The majority of them were spouse (80%). Table 1 also presented the demographic characteristics of individuals with MCI. Most of them were female (53.8%) and married (84.6%). All of them were retired, with a mean age of 75.2 (±6.6) years (range from 62 to 87). More than half of them had educational level at high school or above (54%).

**Table 1:** Characteristics of in-depth Interview Caregivers and Individuals with MCI.

Characteristic	Individuals with MCI		Caregivers	
	N = 13	%	N = 13	%
*Age (years)*				
≤54	0	0.0	2	15.4
55–64	1	7.7	1	7.7
65–74	7	53.8	7	53.8
75–84	4	30.8	2	15.4
≥85	1	7.7	1	7.7
*Gender*				
Male	6	46.2	6	46.2
Female	7	53.8	7	53.8
*Marital status*				
Single	2	15.4	1	7.7
Married	11	84.6	12	92.3
*Highest education level*				
Illiterate	0	0.0	1	7.7
Middle school graduate	6	46.2	0	0.0
High school graduate	2	15.3	5	38.5
Community college graduate	1	7.7	3	23.1
≥ College degree	4	30.8	4	30.8
*Annual income* (one dollar is equivalent to 6.831 RMB in 2009)				
≤RMB15000	6	46.1	3	23.1
RMB15001–30000	3	23.1	4	30.8
>RM30000	3	23.1	3	23.1
Missing	1	7.7	3	23.1
*Work status*				
Full-time	0	0.0	1	7.7
Part-time	0	0.0	0	0.0
Retired	13	100.0	11	84.6
Rehired after retirement	0	0.0	0	0.0
Unemployed	0	0.0	1	7.7
*Relationship to recipient*				
Spouse	–	–	10	76.9
Children	–	–	2	15.4
Sibling	–	–	1	7.7
Other	–	–	0	0.0

### Thematic Analysis Findings

Three main themes emerged, including: initial recognition of cognitive decline, experience of the diagnosis of MCI, and perception of cognitive decline as a normal part of aging. Some sub-themes were identified under each of the main themes (Table 2).

**Table 2:** Summary of the Themes and Subthemes.

Response category	Specific responses
Initial recognition of cognitive decline	
Initial signs of cognitive decline	Memory loss, behavior changes
Initial diagnosis of MCI or dementia	Reminders
Experience of the diagnosis of MCI	
Treatment and remedies	Medicine doesn’t work, chat with others, protective knowledge
Caregiving experience	keeping a normal routine
Caregivers’ worries	Future
Stigma of the term dementia	Bad “*laonian chidai*”, discrimination, shame
Perception of cognitive decline as a normal part of aging	
Elders’ cognitive decline as natural	Simply this way, normal
No need to hide the disease	Don’t mind,it is only a disease, care more
Still respect individuals with MCI	Being respected as before

### Initial Recognition of Cognitive Decline

Two sub-themes were identified in response to recognition of cognitive decline: initial signs of cognitive decline, and initial diagnosis of MCI.

#### Initial signs of cognitive decline

Although the symptoms of individuals with MCI varied, most participants began the stories with the common sign of memory loss. A spouse stated:

She has displayed the symptoms of cognitive decline for 5–6 years, mainly in memory decline. She would forget the things that she finished just a minute ago and would forget what to do.

In addition, signs of cognitive decline, such as interest change and behavior change, were noticed by the participants too. For example, a spouse commented:

About one year ago, we found he was easy to forget what he was ready to do… Additionally, he had enjoyed riding his bicycle around the town, regarding it as an essential sport, however, now, he doesn’t like it at all… we don’t know the reason why he changed…

Another spouse stated:

In 2007, she began to show signs of memory loss, sometimes with delusion. Later, her social abilities declined, and she even lost interest in going to the senior university to learn electronic keyboarding, something she used to be fond of. Being forgetful, her short-term memory doesn't work well, and she will mess up in the morning and in the afternoon after a nap…

Similarly, a spouse said:

… She used to wash the dirty sheets and clothes herself, and as a routine, she was always in charge of washing dishes, for she didn't think others could wash it cleanly. Yet in the recent six months, she would do half of the dish washing and leave the rest unwashed.

#### Initial diagnosis of MCI

Most participants did not take the individuals with MCI to the memory clinics until they encountered some trigger to seeking care. For example, a spouse stated:

He has a poor memory, but we never talk about it seriously. We did not see a doctor for this until we read the recruitment flyer on the newspaper, so we went to the memory clinics at the Zhongnan Hospital.

Similarly, a sibling stated:

My sister has a poor memory. My mother died of Alzheimer’s disease, and I think maybe she suffers from it too… I took her to the memory clinics at Zhongnan Hospital after I saw the flyer on the newspaper…

### Experience of the Diagnosis of MCI

Four sub-themes were identified in response to attitude towards the diagnosis: treatment and remedies, caregiving experience, caregivers’ worries, and stigma of the Chinese term for dementia.

#### Treatment and remedies

Most participants reflected that none of the medicines prescribed by doctors worked - neither western medicine nor traditional Chinese medicine. A spouse stated:

I feel that the western medicine (e.g. Drag A) has almost no effect on the disease and her condition did not improve.

Similarly, another spouse stated:

We went to Zhongnan hospital in April, 2008, and she was diagnosed with MCI, then she began to take medicine, mainly traditional Chinese medicine, to expand the blood vessels and nurture the brain cells. The doctor prescribed western medicine (e.g. Drug B) too. But they didn't work.

Most participants encouraged their family member who was suffering from MCI to go out to associate with others because they believed that interpersonal interaction, such as chatting with others, would be beneficial for cognitive health. A spouse stated:

… of course, I like him to go out. I take a walk with him every morning and evening. I encourage him to go out to chat with the neighbors. If he always stays alone, his cognitive health will decline more quickly.

Another spouse said:

… If she wants to go out or to associate with others, I am very happy to accompany her. It will be beneficial to her cognitive health.

Similarly, a son commented:

I would like that he go out to chat with others or to exercise. It can be helpful for emotions and mental health…

Since there is no cure treatment, preventive knowledge was expected by a majority of the participants. For example, a sibling said:

This disease, we know, can only be ameliorated. We have searched for some information of the disease on the internet and consulted doctors, and we know there was no effective medicine for cognitive impairment until now… I have a very strong interest in how to prevent the onset of dementia and slow the progression from MCI to dementia… [She hopes to keep in touch with the researchers].

#### Caregiving experience

Most participants depicted their caregiving experience as keeping a normal routine and helping maintain the independence of their family members who were suffering from MCI. A spouse commented:

My main tasks are to prepare meals for him, accompany him to take a walk outside… Basically, he can take care of himself… I do some preparations for him, and he can cook for himself…

Another spouse stated:

We are together every day. My main caregiving is to remind her to take medicine, cook for her, take a walk with her, shop in the vegetable market and watch TV with her… She can do some housework, and most of the housework was done by her…

Similarly, a sibling stated:

In general, she can take care of herself, such as cook, washing, and cleaning… However, if she goes shopping, she may get lost and forget what she wants to do. My main work is to take her out for shopping, accompany her to take a walk…

#### Caregivers’ worries

Some of the participants (especially those who were spouses) expressed their worries about the future: how could their family member with MCI be well cared for when they become too old or weak to continue their caregiving? A spouse stated:

Luckily, now she can basically take care of herself… I am suffering from kidney cancer, having undergone a surgery… Besides, I also suffer from diabetes, prostatitis, and anemia caused by the radiotherapy of kidney cancer… I often worry about how to take care of her if the kidney cancer recurs in me. Even though it won't happen, I am doomed to get older and older day by day, then, who will take care of her and me? I wish that we can live together in a senior house in the future, so that maybe I can take care of her sometimes…

Similarly, a daughter commented:

In general, for me, there has been no burden in caring for my mother till now. If there will be a difficulty, it must be that it will become not easy for me to care of mother as my age grows… For example, sometimes when I help her to take a bath, I feel it is so hard. Usually, I’m very tired after that…

#### Stigma of the chinese term for dementia

In this study, most participants referred to dementia (and AD) by the Chinese term “*laonian chidai*”. Family members were particularly concerned when physicians referred to MCI using the same terminology, as “*laonian chidai*” seemed to arouse more intense concern among the majority of the participants than the illness of MCI itself. For example, a daughter said, “I feel that the terminology ‘*chidai*’ is bad, and to some extent it involves discrimination.” Another daughter stated:

… When the doctor told us that she was diagnosed with MCI, and she has a high risk to develop AD [“*laonian chidai*”], my family and I couldn’t accept the diagnosis of “*chidai*”. Why do doctors call it “*laonian chidai* ”? During this interview, please call it “*jiyili zhang’ai*”, [which means memory decline or memory loss]… The diagnosis was made by the doctor, but we don’t think that she is serious enough to match the diagnosis…

Similarly, a spouse stated:

Generally, I describe the disease as “Alzheimer’s disease” in English rather than “*laonian chidai*” in Chinese. I tell others he will have Alzheimer’s disease, and I tell him [the individual with MCI] that his problem is just memory decline [“*jiyili jiantui*”], otherwise, he will be unhappy…

### Perception of Cognitive Decline as a Normal Part of Aging

Three sub-themes were identified in response to cognitive decline as a normal part of aging: elders’ cognitive decline as natural, no need to hide the disease, maintenance of respect for individuals with MCI.

#### Elders’ cognitive decline as natural

Most participants agreed that it was natural for elders to have cognitive decline, including memory loss. Frequently, they mentioned that “When one gets old, it is simply this way,” “It is normal for elders to lose memory gradually.” A daughter commented:

… She just has some problems with memory. However, for elders, it is the law of nature to be like this… We should respect her more and care for her more …

A spouse stated:

… I think it is normal for her to be like this. Sometimes, she behaves just like a little child and I can understand her.

A son commented:

It is normal for elders to fall ill and show symptoms (like this). Sometimes, his behaviors look funny… We think it will be okay once it passes. There is no need to seek health care or other kinds of help, for it is a natural process and nobody can help…

#### No need to hide the disease

Though most participants showed dislike of the Chinese term “*laonian chidai*”, they stated that there was “no cause for concealment, and did not mind letting other people know about the patient’s disease.” For example, a spouse stated:

I completely don’t mind others knowing about her disease. She suffers from MCI, it is only a disease, and we should care for her more. If others know about her disease, it doesn’t matter. On the contrary, I always tell others about her disease. So, once she does something embarrassing, they can understand her.

Similarly, a daughter stated:

… My family doesn’t mind others knowing about her disease at all. Many elders have memory decline, just like her, it is not a shame for elders to fall ill…

A son stated:

There is no need to hide the disease. It is normal for elders to be like this…

#### Maintenance of respect for individuals with MCI

Based on the common responses regarding cognitive decline as a natural consequence of aging, all participants felt that their family members with MCI “were respected as before.” A daughter stated:

… She is respected by people as before… Of course, she is respected by others. Why shouldn’t people respect her? Not only outsiders but also my family members respect her like before. Son, daughter and grandchildren all respect her and respect her opinions of everything. When outsiders see her, they will greet her politely rather than talking back or dodging her.

A spouse stated:

She is respected by others as before. Outsiders are happy to chat with her…Family members treat her like before as well. She has fallen ill and we should care more about her.

## Discussion

As far as we are aware, our study is one of the first to describe the knowledge of MCI as revealed through family caregivers in China. Similar to the limited studies on family members and caregivers of individuals with MCI in other parts of the world, the common initial sign noticed by family members was memory loss [27]–[29]. However, unlike the reports in the U.S., memory loss did not arouse concern among family caregivers that leads to physician visits [2], [30]. They conveyed the information that they only sought treatment and care when something triggered it, such as seeing a recruitment flyer for a study on cognitive impairment in the newspaper. Though dementia was perceived as an important health issue by the public in the U.S. [18], in China, it was perceived as part of the normal aging process and traditionally aroused little public concern. In urban China, 46% family caregivers of individuals with AD didn’t know where they could seek health care for their family members with AD, with 63.6% elders knowing the incipient symptoms (e.g. memory loss) of MCI and 54.2% elders knowing the prognosis of MCI [10], [31], [32]. When recruitment flyers which involved brief introduction of symptoms, characteristics and prognosis of MCI with detailed recruit information were seen by family members of elders, they would compare the various behaviors of their elderly family member with the symptoms and characteristics of MCI. Then, the recruitment flyers may guide them to take their family members to Zhongnan Hospital to seek health care service.

There is no proven treatment or therapy for MCI till now. As MCI represents a prodromal stage to AD, drugs used to treat AD (e.g. rivastigmine and donepezil) were thought to be probably useful to treat MCI or prevent the progression into AD. However, studies found that medications either failed to stop or slow the progression into AD for individuals with MCI [33] or showed only minor, short-term benefits with significant side effects [34]. In traditional Chinese medicine theories, dysfunctions of kidney, heart, liver, spleen and lung are the main pathogenesis of dementia, and treatment of dementia should be based on the individual’s specific characteristics. Some traditional Chinese medicine therapies, such as acupuncture and herbal treatments, have tried to treat cognitive impairment. However, there is no proven traditional Chinese medicine for MCI or AD even untill now. The lack of an effective medication to cure AD provided an incentive for participants to expect and seek more information on the delay and the prevention of MCI into AD.

In this study, the participants were interviewed when the individuals with MCI had been on treatment for less than a month. Even when all of the interviews and feedbacks were completed in December, 2012, it was less than three months since the individuals with MCI were diagnosed. Though participants in this study implied that none of the medications worked - neither western medicine nor traditional Chinese medicine, it is still possible that the perceived ineffectiveness of the medications could caused by a variety of factors. For example, the medications prescribed by the doctor may need longer period to be particularly effective in the MCI stage, family caregivers may have been expecting too much on the effectiveness of the treatment in their quest to “cure” the patients’ cognitive problem and hadn’t noticed the patient’s subtle slowing of their cognitive decline.

Cognitive impairments are still under-recognized by Chinese health providers. A study conducted in 2008 showed that less than 56% medical staff in major hospitals in China knew the diagnosis criteria of AD [32]. Memory clinics are still an unfamiliar sight in China as they have been established only recently in a few major hospitals in large urban areas. Although a standard way of presenting the diagnosis of MCI was practiced in these memory clinics, many physicians in these memory clinics are more oriented towards prescribing drugs to treat symptoms of memory problems, rather than providing patients and their family with sufficient information on how to manage the symptoms and the types of health promoting behaviors and activities that might reduce the risk or slow the onset of conversion. General physicians in major hospitals do not provide detailed information about health promotion strategies due to the lack of knowledge and for those who are knowledgeable, time constraints. Usually, physicians were busy with the prescriptions, and pharmaceutical treatment usually started once the individuals were diagnosed as MCI without presenting information on the risk of conversion to dementia to the individuals with MCI and their family members. A lack of routine discussions about the conversion from MCI to AD/dementia with the physician and the inadequate knowledge obtained from the instruction of the prescription may further exacerbate participants’ worries about the future.

Meanwhile, physicians in community health care service centers, who are able to have longer and more intensive visits with the patient, should play a more important role in providing health education about MCI, its possible progression and health promoting activities. However, a lack of knowledge of cognitive impairment is still prevalent among them. Furthermore, it is rare for physicians in community health service centers, where most individuals receive care, to recognize and diagnose cognitive impairment. Most health promotion activities in communities provided by physicians in community health care service centers focus on other chronic diseases, such as hypertension and diabetes. Therefore, the primary source of information about cognitive impairment is through physicians in memory clinics in major hospitals, who are difficult to access, due to the current limited availability of health care resources (e.g., trained physicians and memory clinics). This may partially explain the delay from recognition of the disease to physician visits. Therefore, it is important to train physicians to be more knowledgeable about the disease with regard to disease recognition and diagnosis. Also, providing health education about the disease in the community (e.g., community health centers) would also be useful.

Due to few avenues to obtain knowledge about cognitive impairment, worries of the conversion from MCI to AD would spread among family caregivers once one was diagnosed with MCI. Taking the progressive nature of AD into account, family caregivers who played the central role in the home care for the elderly began to worry about future caregiving. Since the birth control policy launched in China in the late 1970s, family size continued to shrink and the number of elderly has kept rising [35]. This problem has been named the “4∶2:1” phenomenon [36]. The estimated increases in the number of elderly who will need dementia care coincides with a dramatic decrease in the number of family members who will be available to provide care [37], [38]. Yet, there are few options available for family caregivers. While it is widely acknowledged that there is a need for additional formal services such as adult day care, respite care, and institutional care services, the formal long-term care system remains in the preliminary stages of development in urban China and most institutional care facilities are not prepared to provide care for elders with cognitive impairment [39]–[41].

As shown in other studies among Chinese Americans, in this study, Chinese family caregivers of individuals with dementia also associated cognitive impairment with normal age related processes [42], [43]. Just like other functional decline among elders, cognitive decline has no treatment offered to prevent its progression until now. This may explain why most participants perceived their family members’ symptoms as normal aging even after their family member was given a diagnosis of MCI. However, in this study, most participants agreed that cognitive decline due to MCI was a disease since it has been diagnosed by doctors. This contradictory perception, combined with a lack of knowledge of the disease condition, may contribute to the delay in seeking out a physician. In one study, only about a half of family caregivers of individuals with AD who recognized the problems brought the demented ones to the memory clinics; 72% of those who did not bring the demented individuals to the memory clinics did not do so because they believed it was part of the normal aging process, and the remaining 20% believed that there was no effective treatment [10].

Traditionally, elders were respected because they are believed to be wiser than other age groups. In this study, family members maintained their respect to the cognitively impaired elderly. However, similar to other studies conducted in China [43]–[47], this study found that the Chinese term for dementia “*laonian chid*ai,” was stigmatizing. Chinese family caregivers of individuals with MCI understood the terminology of the disease more from its literal than biomedical meaning: they associated the term with its cultural incongruity rather than with its neurological symptomatology or etiology. *Chidai* is an adjective which means dull-witted and demented, and the term for AD/dementia, *laonian chidai*, literally translates to “stupid, demented elderly.” It has been reported that the term, *laonian chidai*, significantly increases dementia family caregivers’ risk for depression and results in discrimination of individuals with MCI which is stigmatized as the early stage of AD [46]–[48]. In order to eliminate the discrimination for AD, in October, 2012, the Ministry of Health of China launched a rectification to change “*laonian chidai*” into “*aercihaimeibing*” which is the transliteration name of “Alzheimer's disease”. As the new term for AD is relative professional, popularizing it may need a long period. Also, alternative term is needed for MCI. Some terms, such as aged related cognitive impairment or the transliteration name of “mild cognitive impairment,” needs further discussion.

Family caregivers for individuals with MCI may have different experiences at different stage of caregiving. In this study, most individuals with aMCI were living independently and a few of them receive some activities of daily living (ADL) support due to their older age and frailty. The predominant symptom of individuals with aMCI was memory loss and the interviews were completed soon after the individuals were diagnosed. Therefore, family caregivers in this study reported less experience of burden and stress and they talked more about how they coped with memory loss. One study conducted in the U.S. interviewed 11 spouse caregivers who identified themselves as the primary caregiver for individuals with MCI and found that different themes were revealed. These themes include putting the puzzle pieces together-that there really is something wrong, a downward spiral into a world of silence, consequences to caregivers of living in a world of silence and taking charge of care [20]. Meanwhile, the limitations of this study, such as inclusion of only individuals with aMCI, participants recruited only from memory clinics at one hospital, may influence the representativeness of study results, and the study results may be different because the initial reactions to the diagnosis and treatment can change over time and family caregivers for individuals with MCI may have different experiences in a longer period of time. In addition, comprehensive neurocognitive assessments should be implemented in the future in order to more accurate identify individuals with MCI and their family members.

### Conclusion

This study provided unique data to guide interventions in helping Chinese family members improve their awareness and increase their knowledge of MCI to deal more effectively with MCI diagnosis. The findings suggest that family members’ worries may be relieved by improving family members’ access to accurate knowledge of the disease, and community-based and institutional care services. Physicians in community health care centers require knowledge of cognitive impairment in order to reduce the stigma experienced by individuals with MCI and their family members. Finally, culturally appropriately words are needed for MCI.
